# Supplementary material for: Previous malaria exposure attenuates monocyte-driven inflammation and correlates with modulation of the B cell response
Source: JCI Insight. 2026 Sep 8;11(20):e199206. doi: 10.1172/jci.insight.199206 (PMC13726556; doi:10.1172/jci.insight.199206)
Supplement: Supplementary file 1 [file jciinsight-11-199206-s190.pdf]

Supplementary Material

Table S1. Demographics

Characteristic	Statistic	Number of participants	Primary infected	Previously exposed	Healthy control	P-value
**Related to Figure 2D-E, and Figure 4**						
**Age, (years)**	Median (Range)	72	34 (20, 60)	41 (27, 63)	-	0.060
**Time since last residency in endemic area, (years)**	Median (Range)	41	-	12 (0, 33)	-	
**Cumulative time of residency in endemic area, (years)**	Median (Range)	44	-	26 (4, 41)	-	
**Female sex**	n (%)	72	6 (25%)	11 (23%)	-	>0.9
**CMV positive**	n (%)	67	16 (70%)	43 (98%)	-	0.002
**Related to Figure 3A-B**						
**Age, (years)**	Median (Range)	11	38 (20, 53)	49 (27, 63)	-	0.8
**Time since last residency in endemic area, (years)**	Median (Range)	6	-	16 (1, 32)		
**Cumulative time of residency in endemic area, (years)**	Median (Range)	6	-	24 (18, 39)		
**Female sex**	n (%)	11	1 (25%)	0 (0%)	-	0.5
**CMV positive**	n (%)	11	3 (60%)	6 (100%)	-	0.2
**Related to Figure 5**						
**Age, (years)**	Median (Range)	57	34 (20, 60)	37 (27, 63)	32 (21, 57)	0.8
**Time since last residency in endemic area, (years)**	Median (Range)	28	-	10 (0, 32)	-	
**Cumulative time of residency in endemic area, (years)**	Median (Range)	28	-	26 (15, 39)	-	
**Female sex**	n (%)	57	4 (27%)	4 (14%)	10 (71%)	0.4
**CMV positive**	n (%)	43	10 (67%)	27 (96%)	-	0.015
**Related to Figure 3 and Figure 6 plasma pools**						

**Age, (years)**	Median (Range)	16	41 (27, 53)	45 (27, 63)	-	>0.9
**Time since last residency in endemic area, (years)**	Median (Range)	8		14 (1, 32)		
**Cumulative time of residency in endemic area, (years)**	Median (Range)	8	-	25 (18,39)	-	
**Female sex**	n (%)	16	4 (50%)	1 (13%)	-	0.3
**CMV positive**	n (%)	16	3 (38%)	8 (100%)	-	0.026
**Related to Figure 7**						
**Age, (years)**	Median (Range)	55	35 (20, 60)	40 (27, 57)	-	0.3
**Time since last residency in endemic area, (years)**	Median (Range)	30	-	13 (0, 33)	-	
**Cumulative time of residency in endemic area, (years)**	Median (Range)	33	-	26 (10, 39)	-	
**Female sex**	n (%)	55	6 (30%)	8 (23%)	-	0.7
**CMV positive**	n (%)	55	14 (70%)	34 (97%)	-	0.007

P-values compare primary infected and previously exposed donors only. Continuous variables were analyzed using the Wilcoxon rank-sum test; categorical variables were analyzed using Fisher's exact test. Healthy controls were excluded from statistical comparisons.

Table S2. Flow cytometry panels

Antibodies used for plasma stimulation flow panel			
**Antibodies**	**Clone**	**Source**	**Identifier**
CD56 BB700	NCAM16.2	BD	Cat#566573, RRID:AB_2744430
IgM BB515	G20-127	BD	Cat#564622, RRID:AB_2738869
CD69 PE-Cy7	FN50	BD	Cat#557745, RRID:AB_396851
CD86 PE-Cy5	IT2.2	BioLegend	Cat#305408, RRID:AB_314528
CD307e (FcRL5) PE	509f6	BioLegend	Cat#340304, RRID:AB_2104588
CD3 APC-H7	SK7	BD	Cat#560176, RRID:AB_1645475
CD16 R718	B73.1	BD	Cat#567228, RRID:AB_3683755
CD268 (BAFF receptor) AF647	11C1	BD	Cat#564817, RRID:AB_2744347
CD11c BV786	B-ly6	BD	Cat#568220, RRID:AB_2916848
CD14 BV711	MψP9	BD	Cat#563372, RRID:AB_2744290
CD27 BV650	M-T271	BD	Cat#564894, RRID:AB_2739004
HLA-DR BV510	G46-6	BD	Cat#563083, RRID:AB_2737994
CD64 BV421	10.1	BD	Cat#562872, RRID:AB_2737856
IgD BUV737	IA6-2	BD	Cat#612798, RRID:AB_2870125
CD19 BUV496	SJ25C1	BD	Cat#612938, RRID:AB_2870221
Green fluorescent reactive dye		Invitrogen	Cat#L34970 A
CountBright absolute counting beads		Thermo Scientific	Cat#C36950

Antibodies used for B cell stimulation flow panel			
**Antibodies**	**Clone**	**Source**	**Identifier**
Aqua fluorescent reactive dye		Invitrogen	L34957
CD38 BV421	HIT2	BD	Cat#562444, RRID:AB_11151894
CD27 BV650	M-T271	BD	Cat#564894, RRID:AB_2739004
CD3 PE-Cy5	UCHT1	BD	Cat#300410, RRID:AB_314064
CD14 PE-Cy5	M5E2	BD	Cat#301864, RRID:AB_2860767
CD19 PE-Cy7	HIB19	BD	Cat#560728, RRID:AB_1727438
CD268 (BAFF receptor) AF647	11C1	BD	Cat#564817, RRID:AB_2744347
CD20 APC-H7	2H7	BD	Cat#641405, RRID:AB_1645729

Figure S1

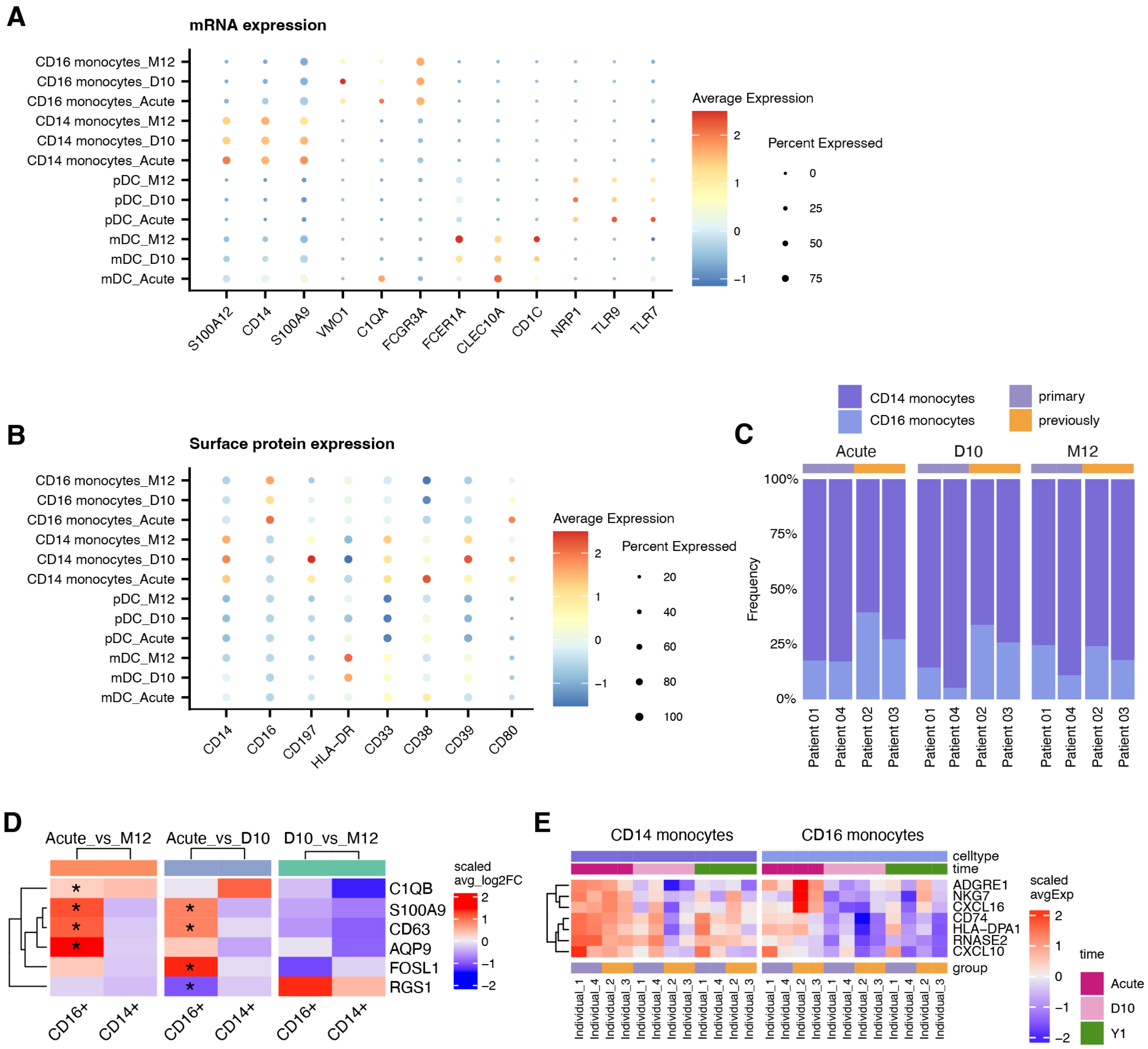

***Figure S1. Gene and protein expression of myeloid cell-specific markers,* related to main Figure 1.** *(**A**) Gene expression in myeloid cells. (**B**) Surface protein expression on myeloid cells. (**C**) Distribution of CD14 and CD16 monocytes for each donor at each time-point. (**D**) Grouped pseudobulk expression of selected genes across monocyte subsets and time points. Statistical analysis for E was performed using EdgeR on individual pseudobulk data with adjusted *P < 0.01. (**E**) Pseudobulk expression of indicated genes for each donor in CD14 and CD16 monocytes.*

Figure S2

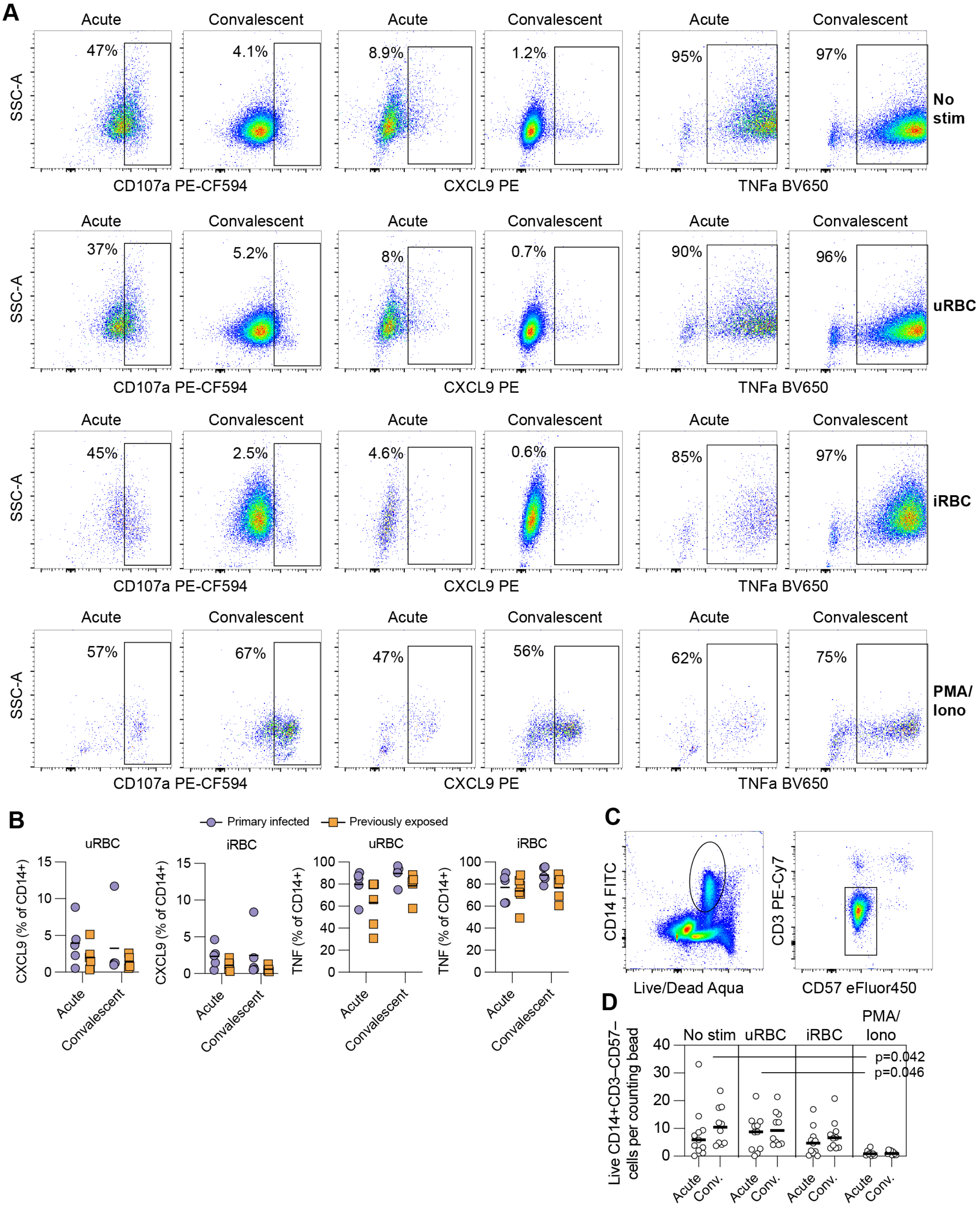

***Figure S2. Gating strategy to assess monocyte CD107a, CXCL9, and TNF-alpha in stimulated PBMCs, related to main Figure 3. (A)** Representative gating strategy to assess the frequency of CD107a, CXCL9, or TNFa-positive Live, CD3–CD57–CD14+ monocytes. Stimulation is indicated on the right with cells receiving no stimulation (media), uninfected RBC (uRBC), infected RBC (iRBC), or phorbol 12-myristate 13-acetate (PMA) and ionomycin (PMA/Iono) stimulation. (**B**) Frequency of CXCL9 and TNFa-positive cells out of total CD14+ monocytes in stimulations with uRBC or iRBC. Statistical analysis was done using a linear mixed effects model followed by Sidak's posttest for correction for multiple testing. Statistical analysis was done using a matched pair two-tailed two-way ANOVA followed by Sidak's posttest. $P < 0.05$ were considered significant. Only significant p-values are*

*shown. (**C**) Gating strategy for Live CD14+ cells (left) with exclusion of contaminating CD3+ and CD57+ cells (right). (**D**) Live CD14+ CD3– CD57– Monocytes per counting bead for individual donors and stimulation condition. Statistical evaluation by two-tailed nested one-way ANOVA with Tukey's correction for multiple testing.*

Figure S3

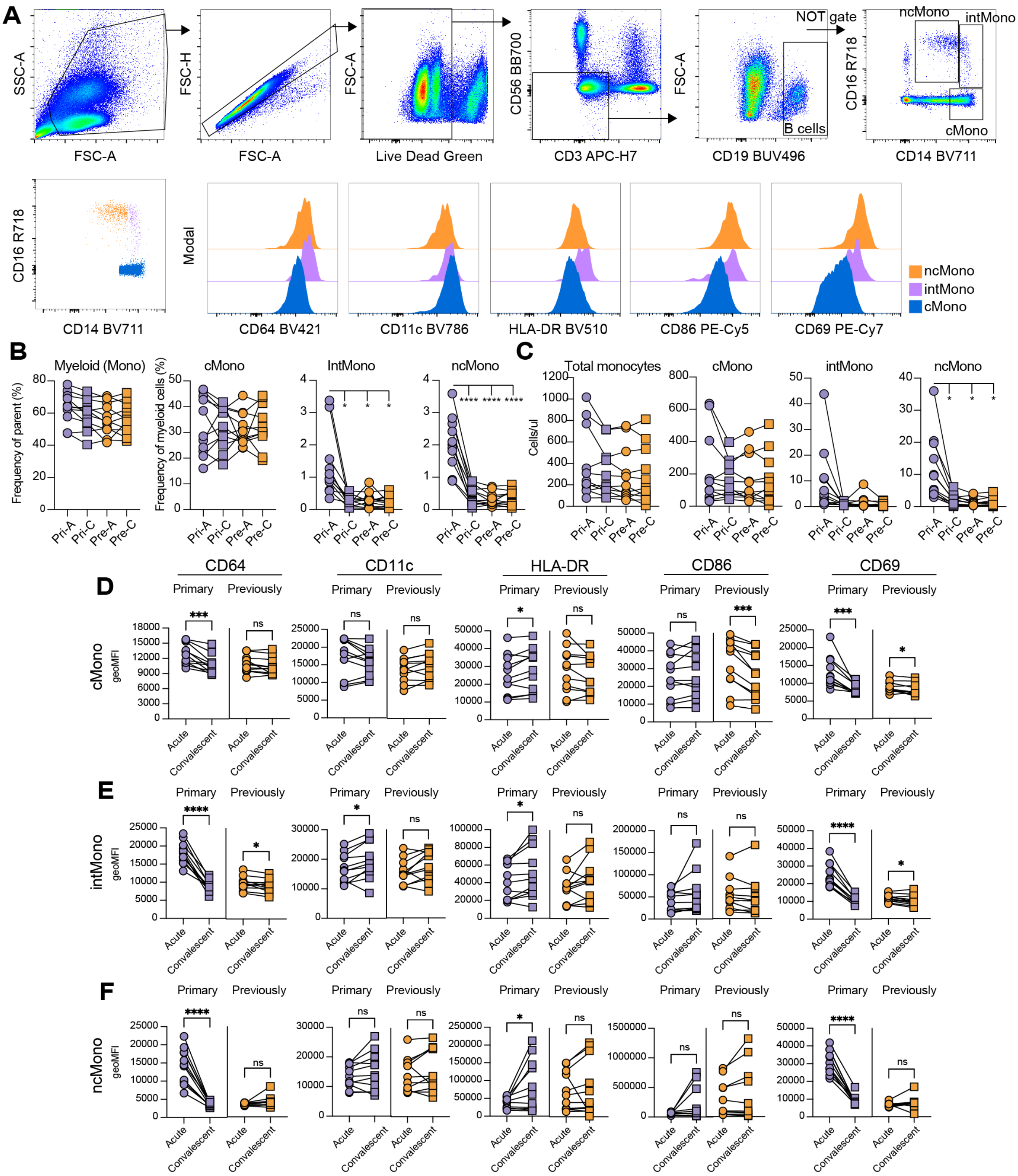

Figure S3. Impact of patient plasma stimulation on monocytes, related to main Figure 3. *(**A**) Gating strategy for monocytes. (**B**) Frequency of parent (live, non-B/T/NK cells). (**C**) Cell counts of total monocytes and monocyte subsets after 24 h PBMC culture with patient plasma pools from primary infected individuals (pri, purple) and previously exposed individuals (pre, orange) at acute malaria (A, circles) and 12 months post-infection (C, convalescent, boxes). (**E-F**) Geometric mean fluorescent intensities (gMFI) for CD64, CD11c, HLA-DR, CD86, and CD69 on (**C**) classical monocytes, (**D**) intermediate monocytes, and (**E**) non-classical monocytes. Statistical analyses for C and D were done using two-way matched pair ANOVA with Geisser-Greenhouse correction followed*

*by Tukey's post-test. Analyses for D-F were done using a two-tailed paired student's t-test. Ns p > 0.05, *p < 0.05, **p < 0.01, ***p < 0.001, ****p < 0.0001.*

Figure S4

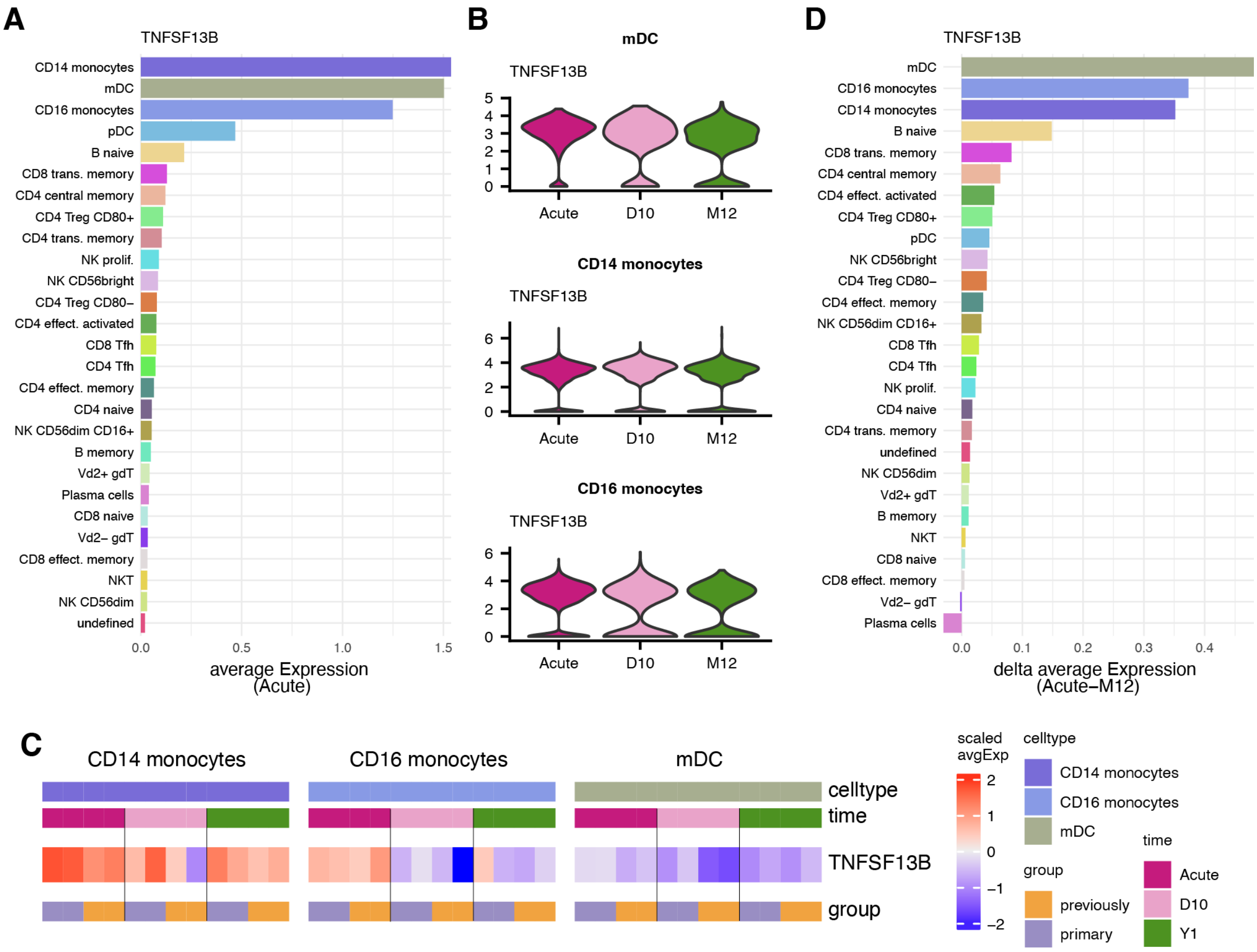

Figure S4. Monocytes and dendritic cells in patients with malaria express the gene encoding for BAFF, related to main Figure 5. *(**A**) The average gene expression of BAFF encoding gene (TNFRSF13) per immune cell subset in PBMCs of four individuals with acute malaria. (**B**) Gene expression of TNFRSF13 over sampling time points, acute (pink), 10 days after (light pink) and 12 months later (green). (**C**) Individual-specific pseudobulk expression of TNFSF13B across myeloid cell subsets and time points. (**D**) Delta average gene expression (Acute-M12) of all immune cell subsets from four individuals.*

Figure S5

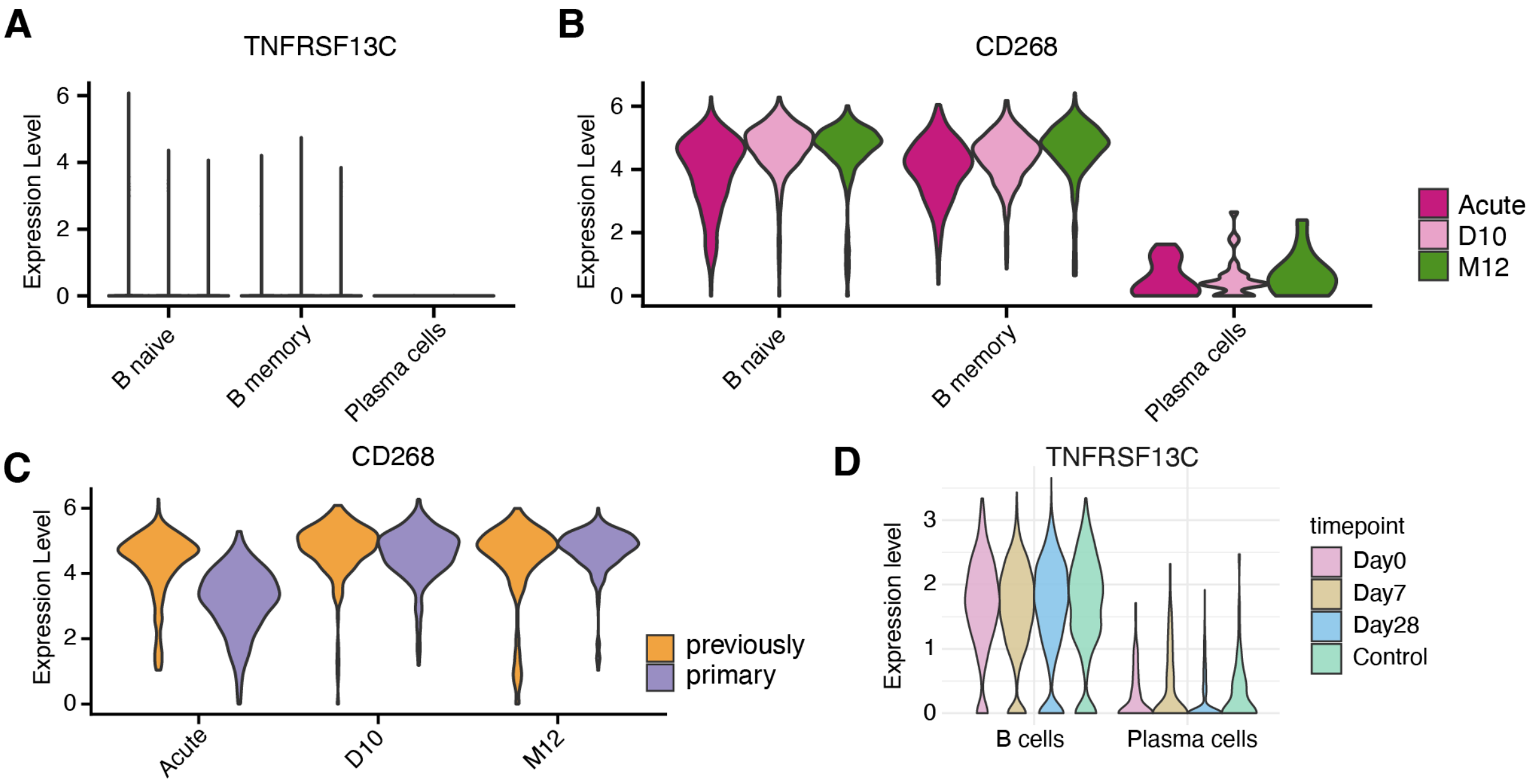

Figure S5. Gene and protein expression on B cells, r*elated to main Figure 5*. *(**A**) Gene expression of TNFRSF13C (BAFF-R) on B cells. (B) CD268 (BAFF-R) protein levels on the cell surface of B cells. (**C**) Differences in CD268/BAFF-R protein levels on naïve B cells during acute malaria and 10 days and 12 months after treatment. (**D**) Repurposed single cell RNA sequencing expression levels from Dooley et al. (25) of TNFRSF13C (BAFF-R) in malaria patients (n = 6 donors) at acute infection (pink), and 7 days (brown), and 28 days (blue) after treatment, and in controls (green, n=2 donors).*

Figure S6

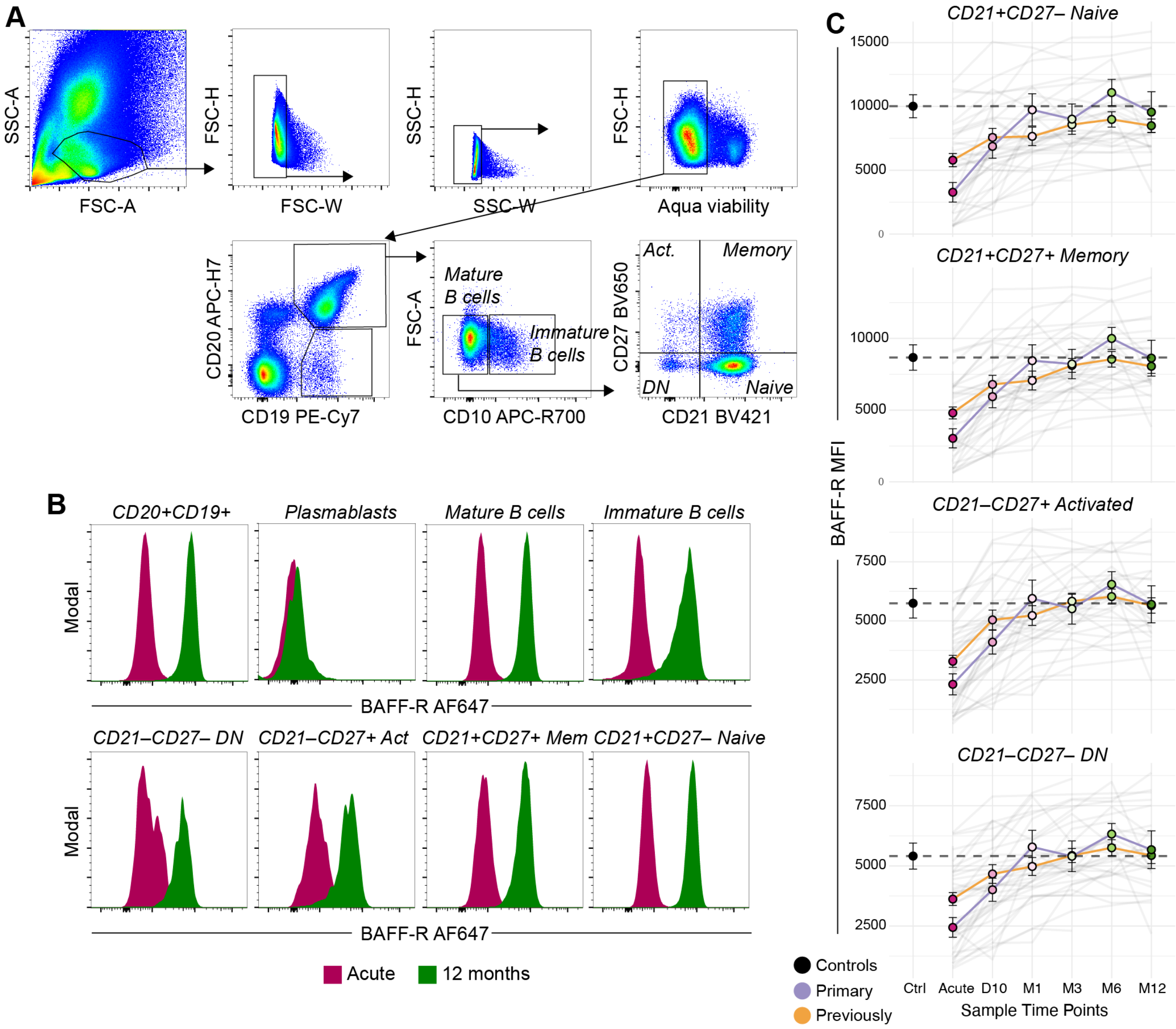

***Figure S6. BAFF-R gating and levels on B cell subsets, related to main Figure 5**. (**A**) Representative gating strategy for B cell subsets. (**B**) Overlay histograms of BAFF expression levels in different B cell subsets at Acute malaria (red) and 12 months after infection (green). (**C**) BAFF-R median fluorescent intensities of primary infected (purple, n=17) and previously exposed (orange, n=34) donors at different time-points following acute malaria for indicated B cell subsets. The mean BAFF-R of healthy controls (black, n=14) is indicated by a dashed line for each cell subset.*

Figure S7

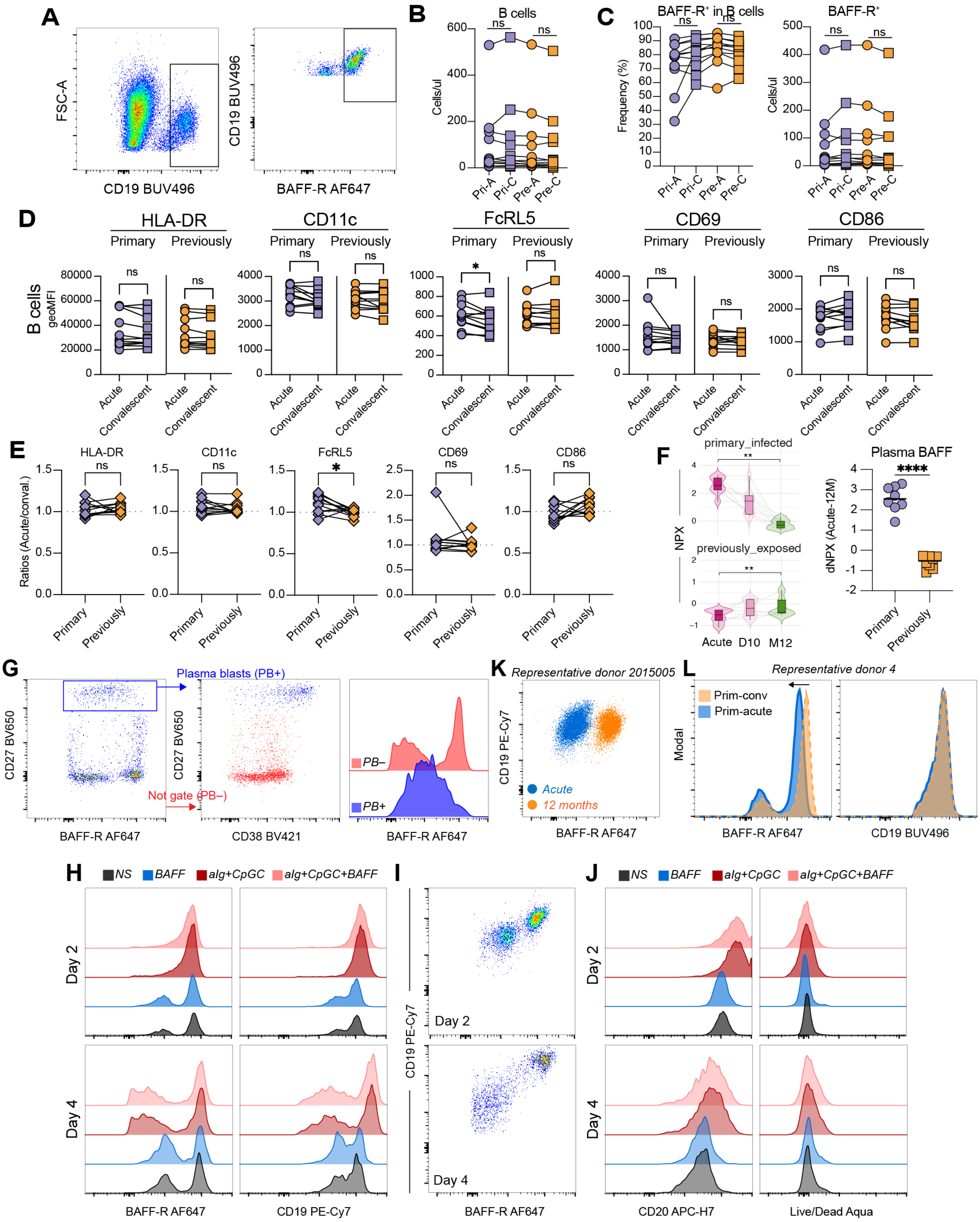

Figure S7. BAFF-R dynamics following in vitro stimulation, **related to main Figure 6.** *(**A**) Representative gating for B cells followed by BAFF-R+ cells. (**B**) CD19+ B cells per microliter. (**C**) Frequency (left) and number (right) of BAFF-R+ cells of B cells. (**D**) Geometric mean fluorescent intensity (geoMFI) of indicated surface proteins on B cells after stimulation with indicated plasma pools for 24 h. (**E**) Ratios (acute/convalescent plasma) for geoMFI marker expression between cells stimulated with primary infected or previously exposed plasma pools. (**F**) BAFF*

*NPX (Acute, 10 days and 12 months) and delta-NPX (Acute–12 months) levels for donors included in the plasma pools. (**G**) BAFF-R versus CD27 at day 4 to gate for plasma blasts seen as CD27 high (PB+, blue) and non-PB (PB–, red) (left panel). Overlay of gated populations to indicate CD38 expression for PB+ and PB– cells (middle panel). Histograms indicating BAFF-R levels for PB+ and PB– cells. (**H**) Histograms indicating BAFF-R and CD19 levels on sorted stimulated B cells after 2 days (top) and 4 days (bottom) of stimulation with type of stimulation indicated by color. (**I**) Pseudocolor dot plots indicating CD19 and BAFF-R levels on stimulated non-PC B cells at day 2 and 4 of stimulation with BAFF alone. (**J**) Histograms indicating CD20 (left) and Live dead Aqua viability dye (right) levels at day 2 (top) and day 4 (bottom) of stimulation. (**K**) CD19 vs BAFF-R ex vivo levels from one representative donor with acute (blue) malaria and at 12 months post treatment (orange). (**L**) Histogram overlay of B cells gated from PBMCs stimulated with acute (orange) or convalescent (blue) plasma from primary infected donors. Arrow indicates shift in BAFF-R high levels. Statistical analyses in B-D was done using two-tailed paired student's t-tests while in E, F it was done using two-tailed unpaired student's t-tests with ns = $p > 0.05$, *$p < 0.05$, **$p < 0.01$, ***$p < 0.001$, ****$p < 0.0001$.*

Figure S8

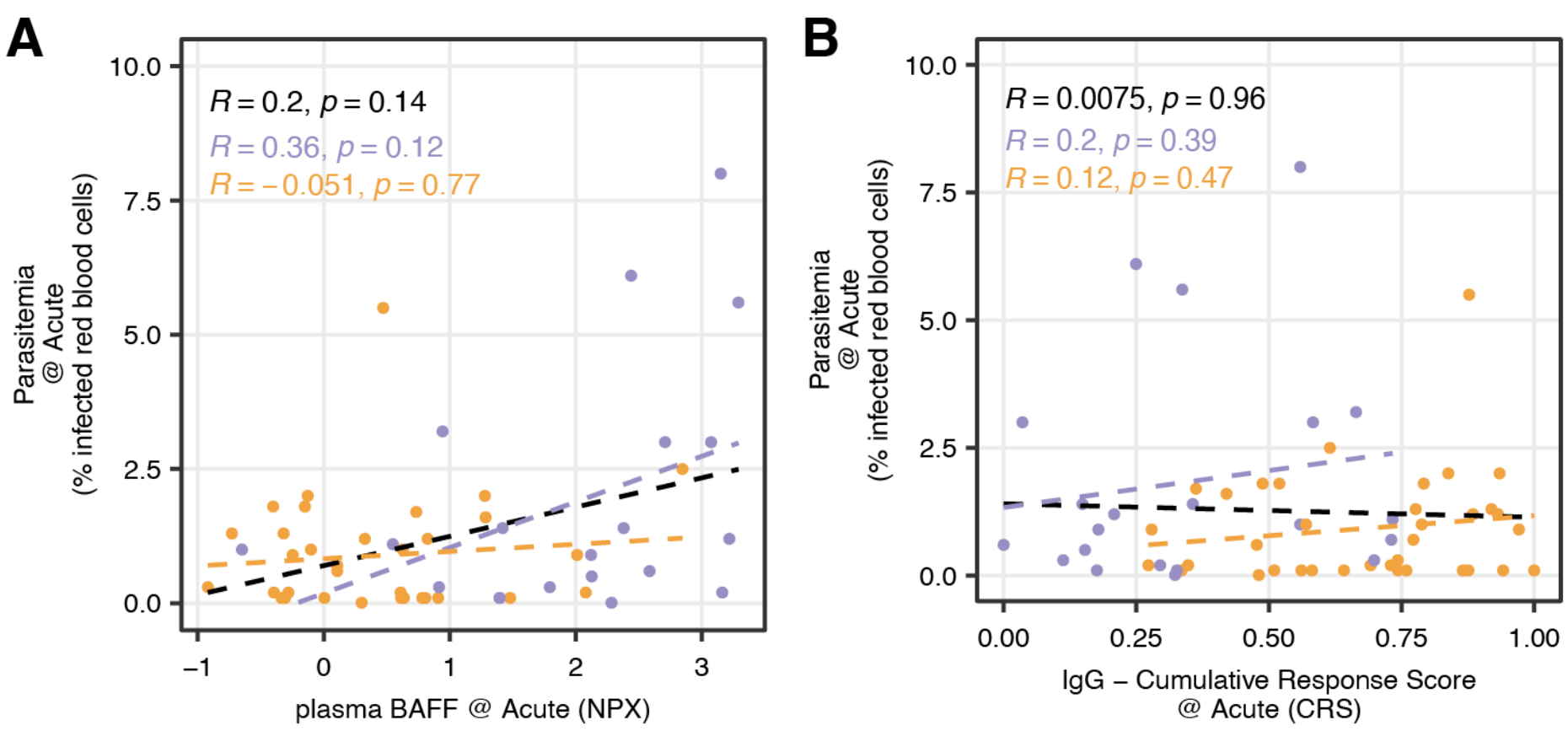

Figure S8. Malaria parasitemia is not correlated with plasma BAFF levels or parasite-specific IgG, related to main Figure 7. *(**A**) Spearman-correlation of parasitemia with (**A**) plasma BAFF levels left and (**B**) cumulative antibody response score (CRS). Dots are colored by status of previous malaria exposure (orange n=35) or primary infection (purple, n=20).*

Figure S9

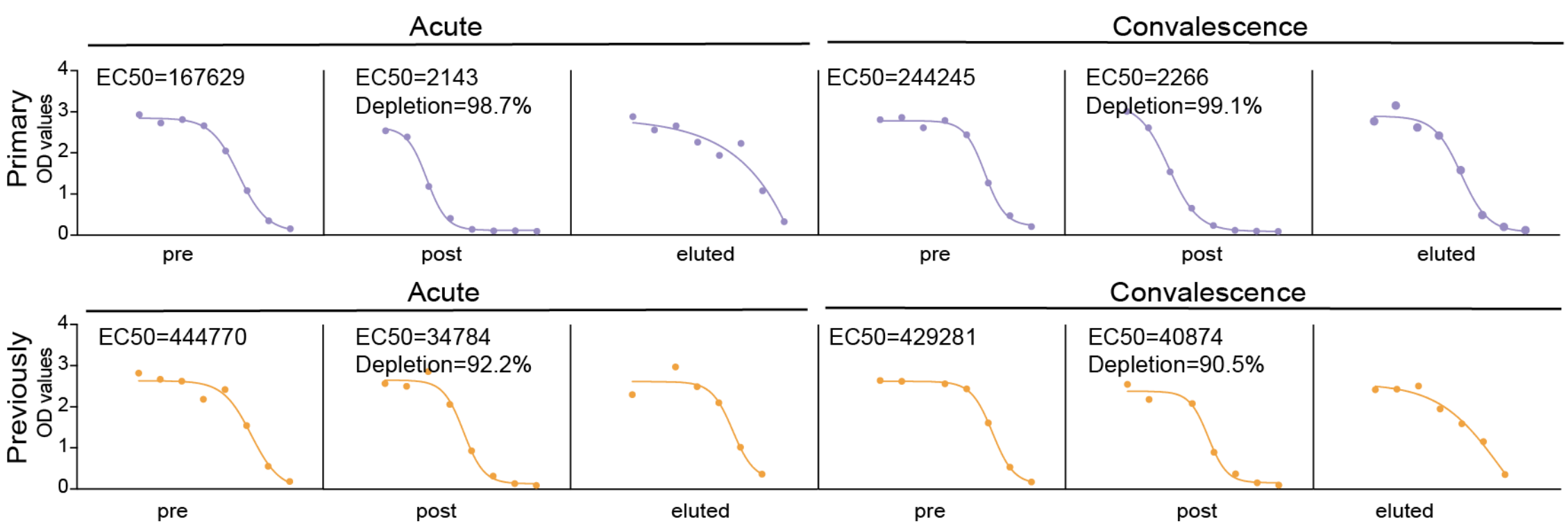

***Figure S9. Plasma pool antibody depletion**. Confirmation of antibody depletion in the four different plasma pools. Depletion was calculated as (1–(EC50-post / EC50-pre))*100.*
